# Impaired Visual Integration in Children with Traumatic Brain Injury: An Observational Study

**DOI:** 10.1371/journal.pone.0144395

**Published:** 2015-12-04

**Authors:** Marsh Königs, Wouter D. Weeda, L. W. Ernest van Heurn, R. Jeroen Vermeulen, J. Carel Goslings, Jan S. K. Luitse, Bwee Tien Poll-Thé, Anita Beelen, Marleen van der Wees, Rachèl J. J. K. Kemps, Coriene E. Catsman-Berrevoets, Jaap Oosterlaan

**Affiliations:** 1 Department of Clinical Neuropsychology, VU University Amsterdam, Amsterdam, The Netherlands; 2 Department of Methods, VU University Amsterdam, Amsterdam, The Netherlands; 3 Institute of Psychology, Department of Methodology and Statistics, Leiden University, Leiden, The Netherlands; 4 Pediatric Surgical Center of Amsterdam, Emma Children’s Hospital Academic Medical Center and VU University Medical Center, Amsterdam, The Netherlands; 5 Department of Pediatric Neurology, VU University Medical Center, Amsterdam, The Netherlands; 6 Department of Pediatric Neurology, Maastricht University, Medical Center, Maastricht, The Netherlands; 7 Trauma Unit, Academic Medical Center, Amsterdam, The Netherlands; 8 Department of Emergency Medicine, Academic Medical Center, Amsterdam, The Netherlands; 9 Department of Pediatric Neurology, Emma Children’s Hospital Academic Medical Centre, Amsterdam, The Netherlands; 10 Merem Rehabilitation Center ‘De Trappenberg’, Huizen, The Netherlands; 11 Department of Rehabilitation, Academic Medical Center, Amsterdam, The Netherlands; 12 Libra Rehabilitation Medicine and Audiology, ‘Blixembosch’, Eindhoven, The Netherlands; 13 Libra Rehabilitation Medicine and Audiology ‘Leijpark’, Tilburg, The Netherlands; 14 Department of Pediatric Neurology, Erasmus Medical Center, Rotterdam, The Netherlands; 15 Emma Children’s Hospital Academic Medical Center, Amsterdam, the Netherlands; University of Wuerzburg, GERMANY

## Abstract

**Background:**

Axonal injury after traumatic brain injury (TBI) may cause impaired sensory integration. We aim to determine the effects of childhood TBI on visual integration in relation to general neurocognitive functioning.

**Methods:**

We compared children aged 6–13 diagnosed with TBI (*n* = 103; M = 1.7 years post-injury) to children with traumatic control (TC) injury (*n* = 44). Three TBI severity groups were distinguished: mild TBI without risk factors for complicated TBI (mild^RF-^ TBI, *n* = 22), mild TBI with ≥1 risk factor (mild^RF+^ TBI, *n* = 46) or moderate/severe TBI (*n* = 35). An experimental paradigm measured speed and accuracy of goal-directed behavior depending on: (1) visual identification; (2) visual localization; or (3) both, measuring visual integration. Group-differences on reaction time (RT) or accuracy were tracked down to task strategy, visual processing efficiency and extra-decisional processes (e.g. response execution) using diffusion model analysis. General neurocognitive functioning was measured by a Wechsler Intelligence Scale short form.

**Results:**

The TBI group had poorer accuracy of visual identification and visual integration than the TC group (Ps ≤ .03; ds ≤ -0.40). Analyses differentiating TBI severity revealed that visual identification accuracy was impaired in the moderate/severe TBI group (P = .05, *d* = -0.50) and that visual integration accuracy was impaired in the mild^RF+^ TBI group and moderate/severe TBI group (Ps < .02, *d*s ≤ -0.56). Diffusion model analyses tracked impaired visual integration accuracy down to lower visual integration efficiency in the mild^RF+^ TBI group and moderate/severe TBI group (Ps < .001, *d*s ≤ -0.73). Importantly, intelligence impairments observed in the TBI group (P = .009, *d* = -0.48) were statistically explained by visual integration efficiency (P = .002).

**Conclusions:**

Children with mild^RF+^ TBI or moderate/severe TBI have impaired visual integration efficiency, which may contribute to poorer general neurocognitive functioning.

## Introduction

Worldwide, an estimated 54–60 million individuals sustain traumatic brain injury (TBI) each year [1], being the leading cause of acquired disability among children and adolescents [2]. Impaired white matter integrity is believed to represent a crucial mechanism in the neuropathology of TBI [3]. Axonal injury causes disconnection of neural networks and is thought to underlie impaired integration of sensory processing after TBI [4]. Visual processing is essential to general neurocognitive functioning [5] and impaired visual integration after TBI may therefore account for daily life difficulties observed in children with TBI.

Axonal injury in TBI is caused by shearing mechanical forces of rapid acceleration and deceleration, followed by secondary biochemical mechanisms involving cytotoxic inflammatory cascades and edema that may result in raised intracranial pressure [6]. A meta-analysis of diffusion tensor imaging studies has shown widespread microstructural white matter abnormalities in children with moderate/severe TBI [7]. Even children with mild TBI have been identified with microstructural white matter abnormalities in the acute phase, while evidence from adults additionally indicates that these white matter abnormalities persist into the chronic phase of recovery [7,8]. These findings indicate that TBI induces white matter damage along the full span of injury severity, with a persisting detrimental impact on white matter integrity.

White matter facilitates the structural connectivity of the brain, allowing the integration of processes originating from specialized brain areas [9–11]. The visual cortex is known to have a high degree of functional specialization [12], thereby crucially relying on visual integration to construct of a full representation of the environment, which is in turn essential for efficient interaction with the environment [5]. The detrimental impact of TBI on white matter integrity -and its associated loss of structural connectivity- is therefore likely to interfere with visual integration. In line with this hypothesis, we showed in a recent meta-analysis of 81 studies that visuospatial functioning is strongly impacted by TBI, and is considered to be more heavily affected than verbal functioning [13]. However, most of the tasks measuring visual functioning used in the literature do not only require visual integration, but also tap a range of other functions including attention, speed of information processing and visuomotor skills [14,15]. To the best of our knowledge, no study to date has attempted to isolate the effects of childhood TBI on visual integration.

We developed the Visual Integration Test to measure the efficiency of goal-directed behavior that is increasingly dependent on visual integration. This computerized test is a sensitive measure of visual processing of identity and location as well as the integration of these processes, with minimal load on motor function and correcting for the potentially confounding influence of processing speed over test conditions. The aim of current study is to elucidate the effects of childhood TBI on visual integration along the full continuum of injury severity. Diffusion model analysis was used to study the contributions of task strategy, efficiency of visual processing and extra-decisional processes (i.e. encoding and response execution) to task performance. Last, we explored the impact of potential visual integration deficits on general neurocognitive functioning as measured by intelligence. We hypothesize that the effects of TBI on the structural connectivity of the brain reduce the efficiency of visual integration, having a negative impact on intelligence.

## Methods

### Participants

#### Sample

This study compared a TBI group of 103 children to a trauma control (TC) group of 44 children with traumatic injury not involving the head, to control for pre-injury risk factors of traumatic injury and psychological effects of hospitalization [16]. All children were retrospectively recruited from a consecutive cohort of three university-affiliated level I trauma centers and three rehabilitation centers in the Netherlands. Inclusion criteria were: (1) age at testing 6–13 years; (2) proficient in the Dutch language; (3) hospital admission with a clinical diagnosis of TBI for inclusion in the TBI group; (4) hospital admission for traumatic injuries below the clavicles for inclusion in the TC group [17], and (5) more than two months post-injury to avoid additional patient burden in the acute phase of recovery. Exclusion criteria were: (1) previous TBI; (2) visual disorder interfering with neurocognitive testing; (3) current neurological condition with known effects on neurocognitive functioning, other than TBI; or (4) clinical diagnosis of dyslexia or dyscalculia, as the task involved in current study required correct identification of numbers. Of all 375 children admitted between October 2009-October 2013 that were eligible for inclusion (TBI and TC: *n* = 232 vs. *n* = 143), 54 were not reached (*n* = 39 vs. *n* = 15) and 137 declined participation (*n* = 68 vs. *n* = 69). Main reasons not to participate were: not interested (25% vs. 32%), no time (22% vs. 22%) or too high a burden on child (8% vs. 16%). Last, 31 children were excluded (TBI: *n* = 6 not proficient in Dutch, *n* = 5 age exceeding criterion, *n* = 1 motor retardation, *n* = 8 dyslexia, *n* = 1 premature termination of participation; TC: *n* = 3 not proficient in Dutch, *n* = 1 previous TBI, *n* = 1 brain tumor, *n* = 1 mental retardation, *n* = 3 dyslexia and *n* = 1 premature termination of participation), while data of six children was not available due to technical failure (TBI: *n* = 1, TC: *n* = 5). The remaining children with TBI (*n* = 103) did not differ from their respective recruitment cohort (*n* = 232) on age (M [SD] = 8.5 [2.0] and M [SD] = 8.7 [2.1], respectively; P = .39) and gender (64% and 58% males, respectively, P = .29). Likewise, the remaining children with TC (*n* = 44) did also not differ from their respective recruitment cohort (*n* = 143) in terms of age (M [SD] = 9.2 [2.2] and M [SD] = 9.6 [2.1], respectively; P = .25) or gender (48% and 63%, respectively; P = .08).

#### Injury severity

Information on injury severity was extracted from medical files and included: (1) diagnosed injuries; (2) the lowest score on the Glasgow Coma Scale (GCS) on the day of admission; (3) admission duration; (4) the presence of risk factors for complicated mild TBI according to the European Federation of Neurological Societies guidelines on mild TBI [18]. These risk factors involved impaired consciousness (GCS = 13–14), focal neurological deficits, persistent vomiting (≥ 3 episodes), post-injury epileptic insults, progressive headache and abnormal CT-scan of the skull or brain [18]. Injury severity was categorized into mild TBI (GCS = 15–13, loss of consciousness [LOC] duration ≤ 30 minutes, post-traumatic amnesia [PTA] duration ≤ 1 hour) without risk factors (mild^RF-^ TBI, *n* = 22), mild TBI with at least one risk factor (mild^RF+^ TBI, *n* = 46), and moderate/severe TBI (GCS = 12–3, LOC duration > 30 minutes, PTA duration > 1 hour; *n* = 35) [19].

### Measures

#### Demographics and clinical diagnoses

Data on gender, age, socio-economic status (SES) and diagnosed psychiatric and learning disorders were collected using a parental questionnaire. SES was defined as the average level of parental education ranging from 1 (no education) to 8 (postdoctoral education) [20].

#### Intelligence

Full-scale IQ (FSIQ) was estimated using a short form of the Wechsler Intelligence Scale for Children-III (including the subtests Vocabulary, Similarities, Block Design and Picture Arrangement), with a previous study indicating excellent validity (*r* = .93) and reliability (*r* = .93) in estimating FSIQ [21].

#### Visual Integration Test

**Fig 1:**
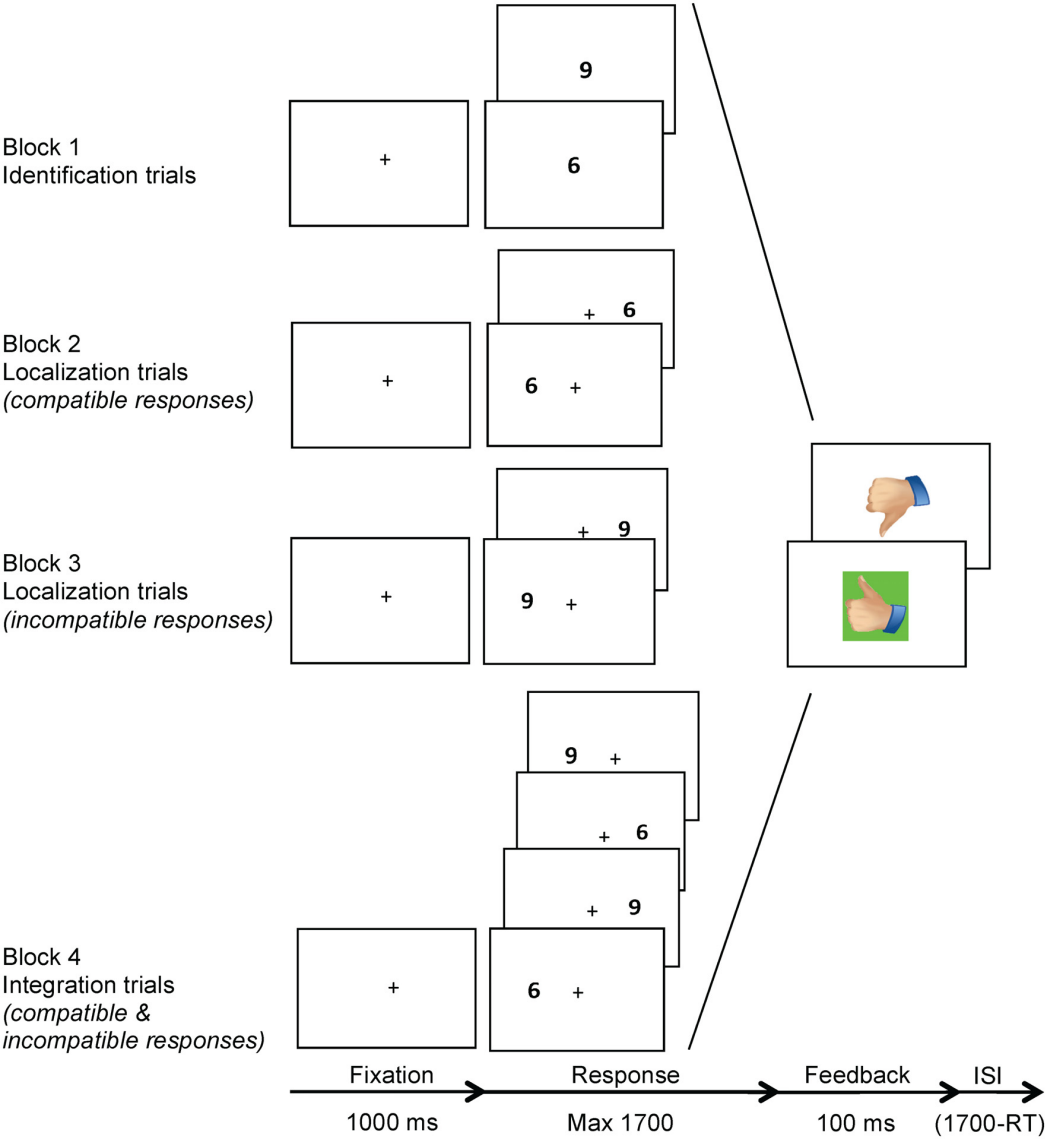
Visual Integration Test procedure. *Note*. The Visual Integration Test consisted of three conditions: the identification, localization and integration conditions, administered in four sequential blocks. The identification condition (block 1) required visual processing of identity: the target was digit ‘6’ or ‘9’ presented in the center of the screen and subjects were required to press the left or right button on a response box, respectively. The localization condition (block 2 and 3) required visual processing of the location of targets that were presented on the left or right side of the screen. In block 2, the target was ‘6’ and the required response was compatible with the target location. In block 3, the target was '9' and the required response was incompatible with the target location. The integration condition (block 4) required processing of the visual identity as well as location of targets presented on the left or right of the screen, measuring visual integration. The targets were either '6' or '9', and they required compatible and incompatible responses, respectively. In other words, a ‘6’ presented on the right side of the screen required a right button response, whereas a ‘9’ presented on the right side of the screen required a left button response, and vice versa. ISI = inter-stimulus interval.

The Visual Integration Test is a computerized paradigm that was designed to measure the efficiency of: (1) identification of visual stimuli, (2) localization of visual stimuli and (3) the integration of these two processes. This test consisted of three conditions: the identification, localization and integration conditions, administered in four sequential blocks (Fig 1). The identification condition (block 1) required visual processing of identity: the target was digit ‘6’ or ‘9’ presented in the center of the screen and subjects were required to press the left or right button on a response box, respectively. The localization condition (block 2 and 3) required visual processing of the location of targets that were presented on the left or right side of the screen. In block 2, the target was ‘6’ and the required response was compatible with the target location. In block 3, the target was '9' and the required response was incompatible with the target location. The integration condition (block 4) required processing of the visual identity as well as location of targets presented on the left or right of the screen, measuring visual integration. The targets were either '6' or '9', and they required compatible and incompatible responses, respectively. In other words, a ‘6’ presented on the right side of the screen required a right button response, whereas a ‘9’ presented on the right side of the screen required a left button response, and vice versa. Dependent variables were mean reaction time (MRT) and accuracy in the three conditions.

#### Diffusion model

In case effects of TBI were observed on MRT or accuracy, we used the diffusion model in order to pinpoint deficits underlying impaired performance on visual identification, localization or integration. The diffusion model is an extensively studied model that allows to distinguish between the effects of *boundary separation* (i.e. task strategy), *drift rate* (i.e. efficiency of visual processing) and *non-decision time* (i.e. extradecisional processes involving encoding and motor response execution) on task performance [22]. The model has been successfully applied to a multitude of different paradigms [23], ranging from relatively simple tasks like lexical decision tasks [24], to more complex switching tasks [25,26]. The diffusion model states that during a task, noisy information accumulates towards one of two response choices until a threshold (i.e. boundary) is reached, after which a response is initiated. Information accumulation starts at *starting point z* and ends when one of the response boundaries is reached. *Boundary separation* described the distance between response boundaries, reflecting the amount of information required to initiate a response. *Boundary separation* depends on the ratio between RT and accuracy (i.e. speed-accuracy trade-off), where higher values indicate a more conservative response strategy (slow but accurate responding) while lower values indicate a more risky strategy (fast and inaccurate responding). *Drift rate* describes the speed and quality of information accumulation, where higher values indicate more efficient processing (fast and accurate) and lower values indicate less efficient processing (slow and inaccurate). *Non-decision time* refers to processes involved in a task other than the decision process, mainly involving encoding and motor response execution. Higher values of non-decision time indicate slower non-decisional processes, while lower values indicate faster non-decisional processes.

### Procedure

The families of eligible children were sent an information letter and contacted by telephone two weeks later. Participating children were administered the Visual Integration Test by trained examiners in a quiet room while parents filled out the questionnaire in a waiting room. The Visual Integration Test was administered during approximately 15 minutes in four sequentially presented blocks: the first block consisted of 30 identification trials, the second and third block of 30 localization trials each and the third block of 60 integration trials. Visually presented feedback on performance was provided following each response throughout the test (Fig 1).

### Ethics statement

This study was approved for all participating centers by the VU University Medical Centre medical ethical committee (NL37226.029.11) and conducted in accordance with the declaration of Helsinki [27]. Parents and participating children aged > 11 years provided written informed consent for participation.

### Statistical Analysis

Statistical analyses were performed using SPSS 22.0. Dependent variables were screened for outliers (-3.29 > z-score > 3.29) which were rescaled to a value one unit extremer than the most extreme non-outlier [28]. There were no missing data. To address group comparability, the TBI group, TBI severity groups and TC group were compared on demographics, injury-related variables and the prevalence of clinical diagnoses using ANOVA and chi-square tests, where appropriate. To assess intelligence after TBI, the TBI and TC group were compared on FSIQ using ANOVA.

Regarding Visual Integration Test performance, trials with very fast RTs suspected of anticipatory behavior (RT < 250 ms) or very slow RTs suspected of distracted behavior (individual outliers at z-score > 3.29), were removed for each subject in each condition. Next, MRT and accuracy were separately analyzed using repeated measures ANOVA with task condition as within-subject variable (identification, localization and integration condition) and group as between-subject variable (TBI and TC group). When a significant interaction between group and task condition was found, the TBI and TC groups were further compared in each task condition separately. When a significant main effect of group was found for a certain task condition, the diffusion model was applied to the RT and accuracy distributions of each participant in that condition using *fast-dm* [29]. The resulting measures, involving *boundary separation* (i.e. task strategy), *drift rate* (i.e. visual processing efficiency) and *non-decision time* (i.e. extra-decisional processes involving encoding and motor response execution) were compared between groups using ANOVA to track down the observed effects of TBI on RT and/or accuracy. In diffusion modeling, *starting point* z was fixed to the center between the two response boundaries, assuming no bias towards one of two responses as the side of the correct or incorrect responses was randomly assigned [26]. The variability parameters were fixed to zero to prevent overfitting [30], while model fit was assessed using the P-value of the Kolmogorov-Smirnov test [29].

If a main effect of group on any dependent variable was obtained, this variable was subjected to: (1) a Pearson correlation analysis with time since injury in the TBI group to explore recovery effects; and (2) follow-up analyses differentiating for TBI severity using a linear polynomial contrast (TC, mild^RF-^ TBI, mild^RF+^ TBI and moderate/severe TBI) and least square difference (LSD) post hoc tests. Last, the role of potential deficits in visual identification, visual localization and visual integration for general neurocognitive functioning after pediatric TBI was investigated using bootstrapping mediation models [31]. Visual Integration Test variables for which group differences were observed, were inserted as mediators of the relation between group membership (TBI-TC group) and FSIQ. As FSIQ is adjusted for age, we also age-adjusted Visual Integration Test variables using linear regression. Analyses were two-tailed, α was set at .05 and effect sizes were expressed as Cohen’s *d*.

## Results

### Patient Characteristics

Patient characteristics regarding demographics, injury severity and clinical diagnoses, and relevant group comparisons are provided in Table 1. Comparisons between the TBI group (*n* = 103) and TC group (*n* = 44) on demographic variables revealed no differences, except for lower SES in the TBI group. As expected on basis of the inclusion criteria, the TBI group had longer hospitalization duration, higher prevalence of CT-scanning and lower prevalence of extracranial fractures and orthopedic surgery than the TC group. The TBI group and TC group did not differ on the prevalence of psychiatric or learning disorders.

**Table 1:** Demographics, injury-related variables, clinical diagnoses and intelligence in the TBI, TC and TBI severity groups.

	Group		Contrast		TBI Severity			Contrasts^a^
	TBI	TC	P	Cohen’s *d*	Mild^RF-^ TBI	Mild^RF+^ TBI	Moderate/Severe TBI	
*n*	103	44			22	46	35	
*Demographics*								
Males, *n* (%)	59 (57)	21 (48)	.29	-	11 (50)	18 (61)	15 (57)	NS
Age at testing in y, M (SD)	8.7 (2.0)	9.3 (2.2)	.12	-0.29	8.5 (2.0)	8.8 (2.0)	8.8 (2.0)	NS
SES, M (SD)	5.3 (1.3)	5.9 (1.1)	**.003**	-0.54	5.2 (1.3)	5.3 (1.2)	5.2 (1.3)	TC > 1,2,3
*Injury-related information*								
Age at injury in y, M (SD)	6.9 (2.3)	7.7 (2.3)	.07	-0.33	6.8 (2.3)	7.1 (2.3)	6.8 (2.5)	NS
Lowest GCS, M (SD)	12.5 (3.5)	-	-	-	15.0 (0.0)	14.6 (0.7)	8.2 (2.8)	3 < 1,2
Hospital admission in *d*	8.3 (19.0)	2.5 (1.9)	**.04**	0.37	1.9 (0.3)	3.4 (2.9)	18.8 (30.1)	3 > TC,1,2
Time since injury in y, M (SD)	1.8 (1.1)	1.6 (0.8)	.34	-0.17	1.7 (1.0)	1.7 (1.0)	1.9 (1.3)	NS
Range	0.3–5.4	0.4–3.5			0.5–3.8	0.3–4.4	0.4–5.4	
Extracranial fracture, *n* (%)	17 (17)	32 (73)	**< .001**		1 (5)	8 (17)	8 (23)	TC > 1,2,3
>1 Extracranial fractures, *n* (%)	7 (7)	4 (9)	.63		0 (0)	3 (7)	4 (11)	NS
CT-scan	83 (81)	1 (2)	**< .001**		9 (41)	41 (89)	34 (97)	2,3 > 1 > TC
Cranial fracture, *n* (%)	36 (35)	-			0 (0)	15 (33)	21 (60)	3 > 2 > 1
Intracranial pathology, *n* (%)	39 (38)	-			0 (0)	15 (33)	24 (69)	3 > 2 > 1
Orthopedic surgery, *n* (%)	9 (9)	35 (80)	**< .001**		1 (5)	7 (15)	1 (3)	TC > 1,2,3
Neurosurgery, *n* (%)	12 (12)	-			0 (0)	0 (0)	12 (34)	3 > 2,1
*Diagnosed conditions*								
Psychiatric disorder, *n* (%)	8 (8)	0 (0)	.06		1 (5)	5 (11)	2 (6)	2 > TC
Premorbid ADHD, *n* (%)	4 (4)	0 (0)	.19		0 (0)	3 (7)	1 (3)	NS
Learning disorder, *n* (%)	1 (1)	0 (0)	.51		0 (0)	0 (0)	1 (3)	NS
*Intelligence*								
FSIQ, M(SD)	98.4 (15.9)	105.8 (14.5)	**.009**	-0.48	101.7 (16.9)	97.8 (15.8)	97.3 (15.7)	TC > 2,3

*Note*. TBI = traumatic brain injury; TC = traumatic control; M = mean; SD = standard deviation; SES = socio-economic status, GCS = Glasgow Coma Scale, CT = computerized tomography, FSIQ = full-scale IQ; NS = not significant

^a^TC = traumatic control; 1 = mild^RF-^ TBI; 2 = mild^RF+^ TBI; 3 = moderate/severe TBI.

Group comparisons differentiating TBI severity also revealed no differences on demographic variables, except for lower SES in all TBI groups as compared to the TC group (Ps ≤ .03). The moderate/severe TBI group had longer hospital admission, lower GCS scores and more neurosurgery as compared to all other groups (Ps ≤ .04). CT-scans were more prevalent in the moderate/severe TBI group and mild^RF+^ TBI group than in the mild^RF-^ TBI group, and in turn more prevalent in the mild^RF-^ TBI group as compared to the TC group (Ps < .001). The available CT-scans revealed progressively more cranial fractures and intracranial pathology in the mild^RF-^, mild^RF+^ and moderate/severe TBI groups (Ps ≤ .01). Differences in the prevalence of clinical diagnoses only reached significance for the higher prevalence of psychiatric conditions in the mild^RF+^ TBI group as compared to the TC group (P = .02).

### Intelligence

FSIQ was lower in the TBI group than the TC group. Follow-up analysis revealed a linear effect of TBI severity on FSIQ (P = .008), reflecting that increasing TBI severity was related to poorer intelligence. More specifically, FSIQ was significantly lower in the mild^RF+^ TBI group (P = .02, *d* = -0.54) and moderate/severe TBI group (P = .02, *d* = -0.58) as compared to the TC group. Time since injury was not related to FSIQ in the TBI group (r = -.03, P = .78).

### Visual Integration Test Performance

#### MRT and accuracy

VIT performance in the TBI and TC groups is displayed in Table 2. We found a main effect of task condition on MRT, indicating progressively slower performance in the localization, identification and integration conditions, respectively. No interaction between group and task condition was found on MRT, indicating that TBI did not differentially affect processing speed across task conditions. A main effect of group was found, reflecting overall slower performance in the TBI group as compared to the TC group. Follow-up analysis revealed a linear effect of TBI severity on overall MRT (P = .02), indicating that more severe TBI was associated with increasingly slower task performance. Post-hoc group comparisons only revealed significantly slower task performance in the moderate/severe TBI group than in the TC group (P = .01, *d* = 0.57).

**Table 2:** MRT and accuracy of Visual Integration Test performance in the TBI and TC groups.

	Group		Condition		Condition x Group		Group		Contrasts	
	TBI	TC	F	P	F	P	F	P	P	Cohen’s *d*
*n*	103	44								
*MRT*			660.4	**< .001**	0.4	.64	4.7	**.03**		
Identification, M (SD)	706 (188)	650 (167)								
Localization, M (SD)	604 (177)	532 (150)								
Integration, M (SD)	982 (206)	901 (254)								
*Accuracy*			66.6	**< .001**	4.2	**.02**	7.8	**.006**		
Identification, M (SD)	0.92 (0.07)	0.95 (0.05)							**.03**	-0.40
Localization, M (SD)	0.97 (0.04)	0.98 (0.02)							.21	-0.22
Integration, M (SD)	0.86 (0.12)	0.91 (0.09)							**.006**	-0.50

*Note*. TBI = traumatic brain injury; TC = trauma control; MRT = mean reaction time; M = mean; SD = standard deviation.

The main effect of task condition on accuracy reflected progressively poorer performance in the visual localization, identification and integration conditions, respectively. Group interacted with task condition on accuracy, suggesting differential effects of TBI on visual identification, localization and integration. Subsequent analyses on separate conditions revealed lower accuracy in the TBI group as compared to the TC group in the identification condition and the integration condition. In the TBI group, time since injury was not related to the accuracy of visual identification or visual integration (*r*s < .07, Ps > 49).

**Fig 2:**
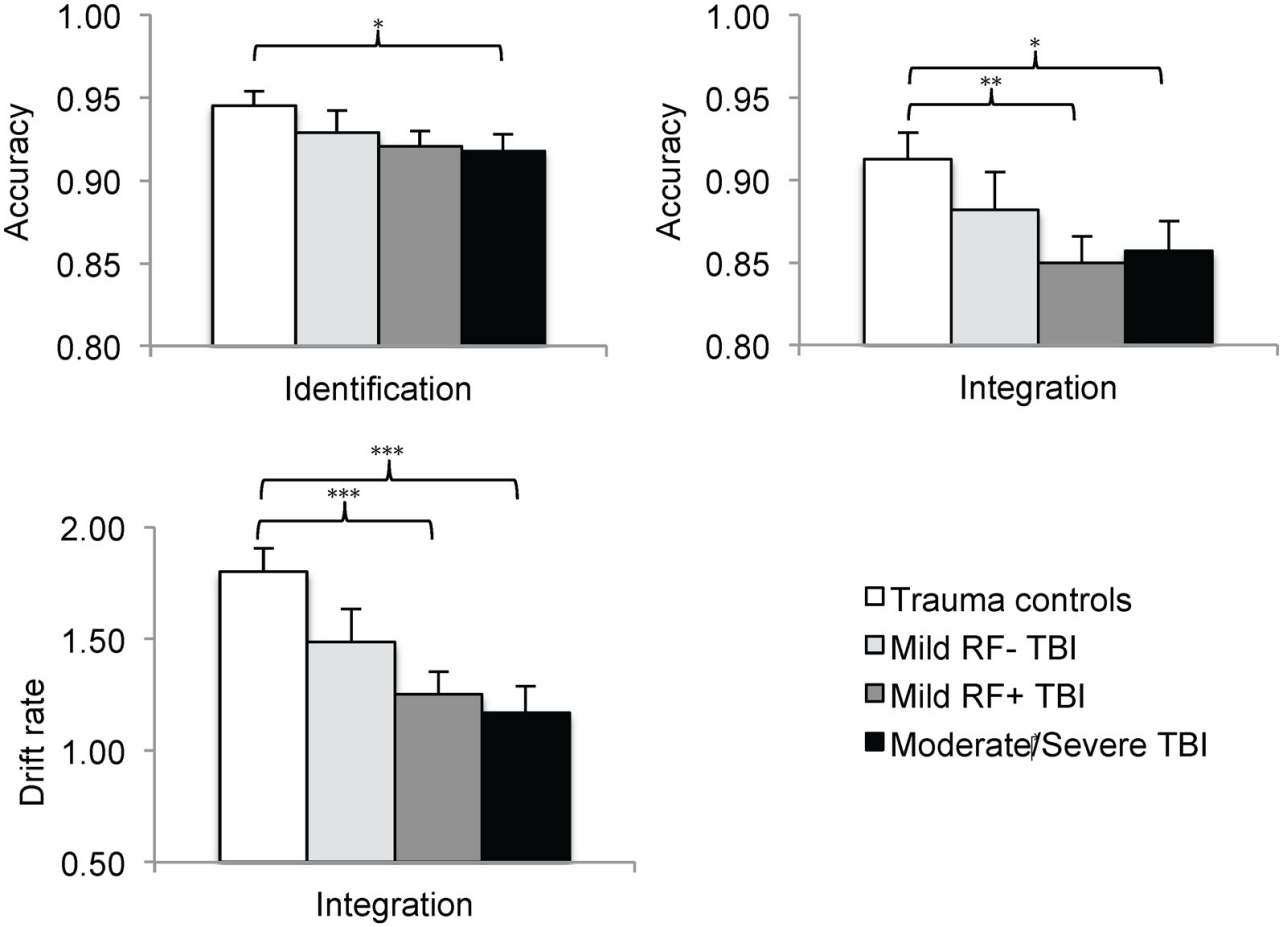
Analyses differentiating TBI severity on visual identification accuracy, visual integration accuracy and visual integration *drift rate*. *Note*. *P < .05 **P < .01 ***P < .001.

Follow-up analyses differentiating TBI severity (displayed in Fig 2) revealed linear effects of TBI severity on accuracy in both the identification condition (P = .04) and the integration condition (P = .01). These findings indicate that more severe TBI was associated with increasingly poorer identification accuracy and integration accuracy. Post-hoc analyses in the identification condition only revealed lower accuracy in the moderate/severe TBI group (P = .05, *d* = -0.50) as compared to the TC group. Post-hoc analyses in the integration condition revealed lower accuracy in the mild^RF+^ TBI group (P = .006, *d* = -0.60) and moderate/severe TBI group (P = .02, *d* = -0.56) than in the TC group.

#### Diffusion model analysis

Diffusion model analysis (Table 3) was used to track down the observed effects of TBI on visual identification accuracy and visual integration accuracy to the following aspects of task performance: *boundary separation* (i.e. task strategy), *drift rate* (i.e. visual processing efficiency) and/or *non-decision time* (i.e. extra-decisional processes involving encoding and motor response execution). With regard to visual identification, the TBI and TC groups did not differ on any of the diffusion model parameters. In contrast, comparisons of diffusion model parameters for visual integration revealed lower visual integration *drift rate* in the TBI group than in the TC group. Time since injury was not related to visual integration *drift rate* in the TBI group (r = .04, P = .70).

**Table 3:** Diffusion model analysis of visual identification and visual integration in the TBI and TC groups.

	Groups		Contrasts	
	TBI	TC	P	Cohen’s *d*
*n*	103	*44*		
*Identification*				
Boundary separation, M (SD)	1.47 (0.42)	1.39 (0.37)	.26	0.20
Drift rate, M (SD)	2.06 (0.97)	2.32 (0.87)	.13	-0.28
Non-decision time, M (SD)	0.39 (0.12)	0.38 (0.12)	.76	0.06
*Integration*				
Boundary separation, M (SD)	1.66 (0.30)	1.68 (0.42)	.68	-0.08
Drift rate, M (SD)	1.27 (0.56)	1.80 (0.96)	**< .001**	-0.76
Non-decision time, M (SD)	0.52 (0.14)	0.50 (0.17)	.*43*	0.14

*Note*. TBI = traumatic brain injury; TC = trauma control; MRT = mean reaction time; *d* = Cohen’s d.

Follow-up analysis on visual integration *drift rate* (Fig 2) revealed a linear effect of TBI severity, indicating that more severe TBI was related to increasingly lower integration *drift rate*. Group differences on visual integration *drift rate* assessed by post-hoc analysis reached significance between the TC group and the mild^RF+^ TBI group (P < .001, *d* = -0.73) and between the TC group and moderate/severe TBI group (P < .001, *d* = -0.81), indicating that mild^RF+^ TBI and moderate/severe TBI have a negative impact on the efficiency of visual integration processing.

### Mediating role of VIT performance

**Fig 3:**
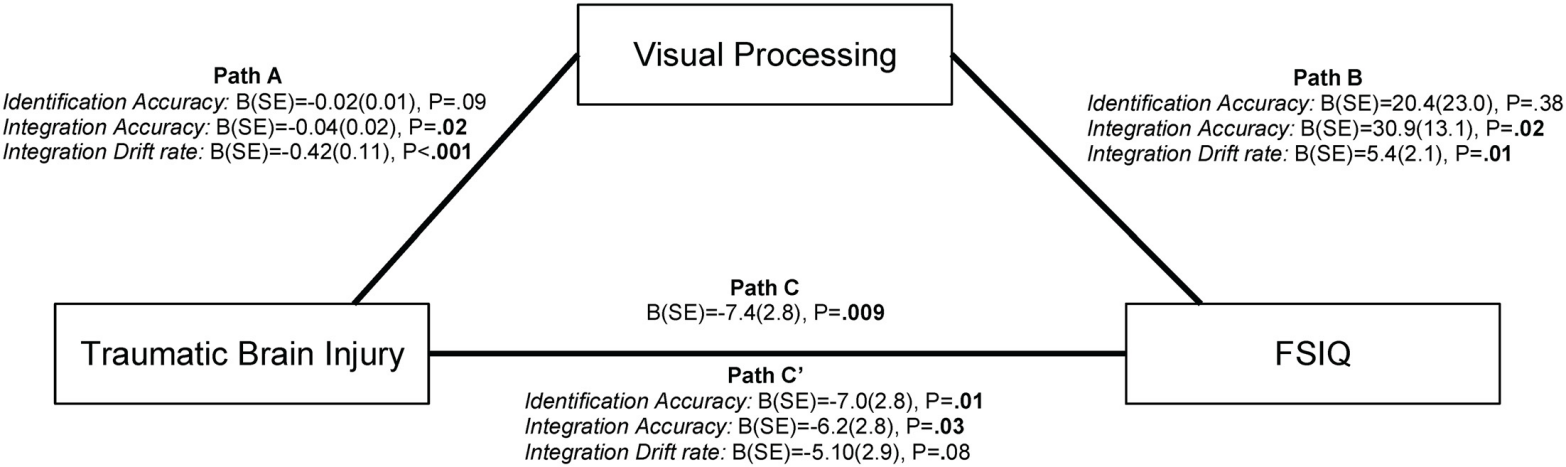
Mediation analysis investigating the roles of visual identification and visual integration in the relation between intelligence impairments and TBI. *Note*. The mediation analysis describes the relations between: (1) group membership and full-scale IQ (FSIQ, Path C); (2) group membership and Visual Integration Test performance (identification accuracy, integration accuracy and integration *drift rate*, Path A); (3) Visual Integration Test performance and FSIQ (Path B); and (4) group membership and FSIQ, corrected for Visual Integration Test performance (Path C’).

We included variables for which effects of TBI were observed (identification accuracy, integration accuracy and integration *drift rate*) in a mediation analysis to investigate the role of visual processing in the relation between TBI and general neurocognitive functioning, as measured by intelligence (FSIQ, Fig 3). As expected, membership of the TBI group (relative to membership of the TC group) was related to lower FSIQ (Path C). Likewise, membership of the TBI group was related to lower integration accuracy as well as lower integration *drift rate* (Path A). Lower integration accuracy and lower integration *drift rate* were in turn related to lower FSIQ (Path B). Importantly, lower integration accuracy partially accounted for the relation between TBI and FSIQ (Path C’; z = -2.5, P = .01), while integration *drift rate* fully explained this relationship (z = -3.1, P = .002). Together these findings suggest that impaired visual integration may contribute to intelligence impairments following TBI. Interestingly, identification accuracy was not related to FSIQ (P = .38), emphasizing the specific role of impaired visual integration in the relationship between TBI and intelligence.

### Confounding analysis

Lower SES was observed in all TBI groups relative to the TC group. Visual Integration Test variables with obtained group differences were not related to SES (Ps ≤ .62), in contrast to FSIQ which showed a strong relationship with SES (*r* = .53, P < .001). To explore the role of SES in the reported effects of mild^RF+^ TBI and moderate/severe TBI on FSIQ, the mild^RF+^ TBI and moderate/severe TBI groups were combined and matched on SES to the TC group (1:1, ±1). The reported difference on FSIQ was replicated using the matched groups (S1 Table), indicating that SES did not confound the reported results. Additionally, we excluded children with intracranial pathology or a psychiatric diagnosis from the mild^RF+^ TBI group and replicated all reported group differences with the remaining group (S2 Table), with the single exception of the reported difference in visual identification accuracy. This finding indicates that intracranial pathology or psychiatric diagnoses did not account for the reported effects of mild^RF+^ TBI on FSIQ and the accuracy and *drift rate* of visual integration.

## Discussion

This study investigated visual integration efficiency in a large sample of children with mild to severe TBI relative to children with trauma control injury. The results indicate that children with mild^RF+^ TBI or moderate/severe TBI have impaired efficiency of visual integration, which was furthermore found to statistically explain intelligence impairments as observed in children with TBI. The findings from this study suggest that visual integration impairments may contribute to the detrimental effects of childhood TBI on general neurocognitive functioning, as measured by intelligence.

We hypothesized that impact of TBI on white matter integrity as described in the literature [7,8] would affect visual integration in children with TBI. To test visual integration in children with TBI, we developed the Visual Integration Test to measure the efficiency of visual identification, visual localization and the integration of these processes during goal-directed behavior. The results show that more severe TBI is related to increasingly lower accuracy of visual identification and visual integration. More specifically, children with moderate/severe TBI have decreased accuracy of visual identification, while the accuracy of visual integration is decreased in children with mild^RF+^ TBI and children with moderate/severe TBI. Application of the diffusion model allowed us to determine the contributions of task strategy (i.e. *boundary separation*), efficiency of information processing (i.e. *drift rate*) and extra-decisional processes (i.e. *non-decision time*, involving encoding and motor response execution) to task performance. Diffusion model analysis tracked down the impact of TBI severity on visual integration accuracy to impaired visual integration *drift rate*. More specifically, children with mild^RF+^ TBI and children with moderate/severe TBI have impaired visual integration *drift rate*, reflecting decreased efficiency of information processing during visual integration. Some children in the mild^RF+^ TBI group had intracranial pathology, which potentially could have accounted for the observed effects of mild^RF+^ TBI. However, the reported results were replicated after exclusion of children with intracranial pathology, indicating that children with mild TBI and risk factors for complicated TBI are at risk of persisting visual integration deficits—even when evidence for intracranial pathology is absent.

Based on the premise that effective interaction with the environment relies on visual integration to construct a full visual representation of our surroundings, we also hypothesized that impaired visual integration deficits would in turn affect general neurocognitive functioning. We found support for this hypothesis in mediation analyses showing that impaired visual integration accuracy and visual integration *drift rate* were related to intelligence in children with TBI. In fact, visual integration *drift rate* fully explained the observed intelligence impairments in children with TBI in statistical terms, while visual integration accuracy partly explained intelligence impairments. Importantly, visual identification accuracy was not related to intelligence, further emphasizing that specifically impaired visual integration (and not impaired identification) was related to intelligence after TBI. Although the results from the mediation model do not necessarily imply causal relationships, these findings suggest that impaired visual integration may importantly contribute to intelligence impairment after childhood TBI.

The results from this study indicating that children with TBI have visual integration deficits are in line with the scarcely available literature. One study showed that a small sample of adult patients with acquired brain injury (*n* = 13) had impaired spatial grouping of visual stimuli, thought to be caused by reduced integration of information in the visual cortex after axonal disruption [32]. A functional magnetic resonance imaging study further showed that an increased load on the integration of visual features in an attention task was associated with impaired task performance in a small sample of patients with diffuse axonal injury after TBI (n = 7) [33]. This impaired performance coincided with widespread increases in neural activation, possibly reflecting recruitment of additional brain areas to compensate the detrimental effects of diffuse axonal injury on visual integration capacity. The results of current study further extend these findings by providing evidence supporting that visual integration impairments may contribute to intelligence impairments in children with TBI. This finding is in line with the earlier reported vulnerability of visuospatial aspects of intelligence to the impact of TBI [13]. However, given the diffuse impact of TBI on white matter integrity, [3] we speculate that integration deficits may not be limited to the visual domain but may also apply to the integration of sensory processes across modalities.

This study has some weaknesses. The sample size of the mild^RF-^ TBI group was somewhat limited, in turn reducing statistical power in analyses comparing TBI severity groups. Furthermore, the TBI group had lower SES (as measured by parental education) than the TC group. Although this pre-injury group difference has the potential to confound group comparisons, we showed that SES did not account for the reported results by: (1) revealing that SES was not related to Visual Integration Test variables; and (2) replicating the reported group differences on FSIQ in SES-matched groups. In addition, pre-injury impairments in visual integration and/or intelligence may increase the risk of sustaining traumatic injury (e.g. through increased risk of falling or risky behavior), meaning that children with TBI may be more likely to have pre-injury impairments in visual integration and/or intelligence as compared to typically developing children. We argue that our results are robust to this influence, since it is unlikely that the described pre-injury impairments selectively increase the risk of TBI as compared to the traumatic injuries sustained by children in the trauma control group. Last, the paradigm used had two potentially confounding factors: the visual integration condition differed from the identification and localization conditions in that (1) visual integration required to comply with two rules instead of one; and (2) visual integration involved shifting between compatible and incompatible responses. Consequently, potential TBI-induced deficits in working memory or set-shifting may have confounded the observed visual integration impairment. However, the TBI group and TC group showed no difference in working memory performance (S3 Table). Moreover, Schmitz & Voss [25] showed that the diffusion model captures set-shifting costs in the *non-decision time* parameter, rather than in the *drift rate* parameter. In line with this finding, exploratory analyses revealed that *non-decision time* was larger in the integration condition than in the identification or localization conditions (S4 Table; Ps < .001). Together with the described absence of an effect of TBI on *non-decision time* in the integration condition, these findings indicate that the observed effects of TBI on visual integration were not confounded by deficits in working memory or set-shifting.

In conclusion, the current study indicates that children with mild^RF+^ TBI (even in the absence of intracranial pathology) and children with moderate/severe TBI have impaired efficiency of visual integration. These visual integration impairments were found to statistically explain the relation between TBI and impairments in general neurocognitive functioning, as measured by intelligence. We speculate that reduced white matter integrity may underlie the observed visual integration deficits in children with TBI [3] while impaired visual integration may in turn importantly contribute to intelligence impairments [5]. To our best knowledge, this is the first study attempting to isolate the effects of childhood TBI on visual integration in behavior and to explore the role of impaired visual integration in general neurocognitive functioning. The results of this study support the clinical importance of visual integration impairments after TBI in childhood.

## Supporting Information

S1 Table Replication of reported findings on FSIQ using the SES-matched TBI group. *Note*. TBI = traumatic brain injury; TC = trauma control; FSIQ = full-scale intelligence quotient; M = mean; SD = standard deviation.
(DOCX)

S2 Table Replication of reported findings on the mild RF^+^ TBI group, while excluding children with intracranial injury or psychiatric conditions. *Note*. TBI = traumatic brain injury; TC = trauma control; FSIQ = full-scale intelligence quotient; M = mean; SD = standard deviation.
(DOCX)

S3 Table Working memory in the TBI and TC groups. *Note*. TBI = traumatic brain injury; TC = trauma control; M = mean; SD = standard deviation.
^a^Digit Span score calculated as the number of correct responses multiplied by the maximum correct span, in the forward and backward conditions separately (n = 2 missing data) according to Verburgh L, Scherder EJ a, van Lange P a M, Oosterlaan J. Executive functioning in highly talented soccer players. PLoS One. 2014;9: e91254.
(DOCX)

S4 Table Non-decision time during the Visual Integration Test in the whole sample. *Note*. M = mean; SD = standard deviation.
(DOCX)

## References

[1] Feigin VL, Theadom A, Barker-Collo S, Starkey NJ, McPherson K, Kahan M, et al Incidence of traumatic brain injury in New Zealand: a population-based study. Lancet Neurol. 2013;12: 53–64. 10.1016/S1474-4422(12)70262-4 23177532

[2] Winslade WJ, Brady JS. Confronting traumatic brain injury: Devastation, hope, and healing. Yale University Press; 1999.

[3] Sharp DJ, Scott G, Leech R. Network dysfunction after traumatic brain injury. Nat Rev Neurol. Nature Publishing Group, a division of Macmillan Publishers Limited. All Rights Reserved.; 2014;10: 156–66. 10.1038/nrneurol.2014.15 24514870

[4] Alwis DS, Johnstone V, Yan E, Rajan R. Diffuse traumatic brain injury and the sensory brain. Clin Exp Pharmacol Physiol. 2013;40: 473–83. 10.1111/1440-1681.12100 23611812

[5] Deary IJ. Human intelligence differences: towards a combined experimental—differential approach. Trends Cogn Sci. 2001;5: 164–170. 11287270 10.1016/s1364-6613(00)01623-5

[6] Bigler ED, Abildskov TJ, Petrie J, Farrer TJ, Dennis M, Simic N, et al Heterogeneity of brain lesions in pediatric traumatic brain injury. Neuropsychology. 2013;27: 438–51. 10.1037/a0032837 23876117

[7] Roberts R, Mathias J, Rose S. Diffusion Tensor Imaging (DTI) Findings Following Pediatric Non-Penetrating TBI: A Meta-Analysis. Dev Neuropsychol. 2014;39: 600–637. 10.1080/87565641.2014.973958 25470224 PMC4270261

[8] Aoki Y, Inokuchi R, Gunshin M, Yahagi N, Suwa H. Diffusion tensor imaging studies of mild traumatic brain injury: a meta-analysis. J Neurol Neurosurg Psychiatry. 2012;83: 870–6. 10.1136/jnnp-2012-302742 22797288 PMC3415311

[9] Park H, Friston K. Structural and functional brain networks: from connections to cognition. Science. 2013;342: 579–587. 10.1126/science.1238411 24179229

[10] Damoiseaux JS, Greicius MD. Greater than the sum of its parts: a review of studies combining structural connectivity and resting-state functional connectivity. Brain Struct Funct. 2009;213: 525–33. 10.1007/s00429-009-0208-6 19565262

[11] Varela F, Lachaux JP, Rodriguez E, Martinerie J. The brainweb: phase synchronization and large-scale integration. Nat Rev Neurosci. 2001;2: 229–39. 11283746 10.1038/35067550

[12] Young M. Objective analysis of the topological organization of the primate cortical visual system. Nature. 1992;358: 152–155. 1614547 10.1038/358152a0

[13] Königs M, Engenhorst PJ, Oosterlaan J. Intelligence after traumatic brain injury: meta-analysis of outcomes and prognosis. Eur J Neurol. 2015; 10.1111/ene.12719 25919757

[14] Ginstfeldt T, Emanuelson I. An overview of attention deficits after paediatric traumatic brain injury. Brain Inj. 2010;24: 1123–1134. 10.3109/02699052.2010.506853 20715886

[15] Catroppa C, Anderson V. Children’s attentional skills 5 years post-TBI. J Pediatr Psychol. 2007;32: 354–369. 16840790 10.1093/jpepsy/jsl019

[16] Max J, Koele S, Jr WS. Psychiatric disorders in children and adolescents after severe traumatic brain injury: a controlled study. Child Adolesc Psychiatry. 1998;37: 932–840. 10.1097/00004583-199808000-00013 9695445

[17] Advanced Trauma Life Support Program for Doctors. American College of Surgeons; 7th edition; 2004.

[18] Vos P, Battistin L. EFNS guideline on mild traumatic brain injury: report of an EFNS task force. Eur J Neurol. 2002;9: 207–219. 11985628 10.1046/j.1468-1331.2002.00407.x

[19] Teasdale G, Jennett B. Assessment and prognosis of coma after head injury. Acta Neurochir (Wien). 1976;34: 45–55. 961490 10.1007/BF01405862

[20] Statistics Netherlands. Education Categorization Standard [Standaard onderwijsindeling 2006]. www.cbs.nl. 2006.

[21] Kaufman AS, Kaufman JC, Baijgopal R MJ. Comparison of three WISC-III short forms, weighing psychometric, clinical and practical factors. J Clin Child Psychol. 1996;25: 97–105.

[22] Ratcliff R. A theory of memory retrieval. Psychol Rev. 1978;85: 59.

[23] Wagenmakers E. Methodological and empirical developments for the Ratcliff diffusion model of response times and accuracy. Eur J Cogn Psychol. 2009;21: 641–671.

[24] Zeguers M, Snellings P. Specifying theories of developmental dyslexia: a diffusion model analysis of word recognition. Dev Sci. 2011;14: 1340–1354. 10.1111/j.1467-7687.2011.01091.x 22010894

[25] Schmitz F, Voss A. Decomposing task-switching costs with the diffusion model. J Exp Psychol Hum Percept Perform. 2012;38: 222–250. 10.1037/a0026003 22060144

[26] Weeda W, Molen M van der. A diffusion model analysis of developmental changes in children’s task switching. J Exp Child Psychol. 2014;126: 178–197. 10.1016/j.jecp.2014.05.001 24945684

[27] World Medical Association Declaration of Helsinki: ethical principles for medical research involving human subjects. JAMA. American Medical Association; 2013;310: 2191–4. 10.1001/jama.2013.281053 24141714

[28] Tabachnick BG, Fidell LS. Using Multivariate Statistics: International Edition. Pearson; 2012.

[29] Voss A, Voss J. Fast-dm: A free program for efficient diffusion model analysis. Behav Res Methods. 2007;39: 767–775. 18183889 10.3758/bf03192967

[30] Voss A, Nagler M, Lerche V. Diffusion models in experimental psychology: A practical introduction. Exp Psychol. 2013;60: 385–402. 10.1027/1618-3169/a000218 23895923

[31] Preacher KJ, Hayes AF. SPSS and SAS procedures for estimating indirect effects in simple mediation models. Behav Res Methods Instrum Comput. 2004;36: 717–31. 15641418 10.3758/bf03206553

[32] Kurylo DD, Larkin GB, Waxman R, Bukhari F. Speed of perceptual grouping in acquired brain injury. Exp brain Res. 2014;232: 2899–905. 10.1007/s00221-014-3970-5 24820289

[33] Raja Beharelle A, Tisserand D, Stuss DT, McIntosh AR, Levine B. Brain activity patterns uniquely supporting visual feature integration after traumatic brain injury. Front Hum Neurosci. 2011;5: 164 10.3389/fnhum.2011.00164 22180740 PMC3238543

