# Supplementary material for: Impaired Visual Integration in Children with Traumatic Brain Injury: An Observational Study
Source: PLoS One. 2015 Dec 4;10(12):e0144395. doi: 10.1371/journal.pone.0144395 (PMC4670090; doi:10.1371/journal.pone.0144395)
Supplement: S1 Table — Note. TBI = traumatic brain injury; TC = trauma control; FSIQ = full-scale intelligence quotient; M = mean; SD = standard deviation. (DOCX) [file pone.0144395.s001.docx]

**Table S1. Replication of reported findings on FSIQ using the SES-matched TBI group.**

|  | Groups | |  | Contrasts | |
| --- | --- | --- | --- | --- | --- |
|  | TBI | TC |  | P | Cohen’s *d* |
| *n* | 44 | 44 |  |  |  |
| *Intelligence* |  |  |  |  |  |
| FSIQ, M (SD) | 99.4 (14.9) | 105.8 (14.5) |  | **.04** | -0.44 |

*Note.* TBI = traumatic brain injury; TC = trauma control; FSIQ = full-scale intelligence quotient; M = mean; SD = standard deviation.
