# Supplementary material for: Impaired Visual Integration in Children with Traumatic Brain Injury: An Observational Study
Source: PLoS One. 2015 Dec 4;10(12):e0144395. doi: 10.1371/journal.pone.0144395 (PMC4670090; doi:10.1371/journal.pone.0144395)
Supplement: S2 Table — Note. TBI = traumatic brain injury; TC = trauma control; FSIQ = full-scale intelligence quotient; M = mean; SD = standard deviation. (DOCX) [file pone.0144395.s002.docx]

**Table S2. Replication of reported findings on mild RF^+^ TBI, while excluding children with intracranial injury or psychiatric conditions.**

|  | Groups | |  | Contrasts | |
| --- | --- | --- | --- | --- | --- |
|  | Mild RF^+^ TBI | TC |  | P | Cohen’s *d* |
| *n* | 30 | 44 |  |  |  |
| *Intelligence* |  |  |  |  |  |
| FSIQ, M (SD) | 96.8 (15.7) | 105.8 (14.5) |  | **.01** | -0.61 |
| *Visual Integration Test* |  |  |  |  |  |
| Identification accuracy, M(SD) | 0.95 (0.05) | 0.93 (0.07) |  | .07 | -0.47 |
| Integration accuracy, M(SD) | 0.84 (0.14) | 0.91 (0.08) |  | **.004** | -0.70 |
| Integration *drift rate*, M(SD) | 1.19 (0.55) | 1.80 (0.96) |  | **.004** | -0.67 |

*Note.* TBI = traumatic brain injury; TC = trauma control; FSIQ = full-scale intelligence quotient; M = mean; SD = standard deviation.
