# Supplementary material for: Impaired Visual Integration in Children with Traumatic Brain Injury: An Observational Study
Source: PLoS One. 2015 Dec 4;10(12):e0144395. doi: 10.1371/journal.pone.0144395 (PMC4670090; doi:10.1371/journal.pone.0144395)
Supplement: S3 Table — Note. TBI = traumatic brain injury; TC = trauma control; M = mean; SD = standard deviation. aDigit Span score calculated as the number of correct responses multiplied by the maximum correct span, in the forward and backward conditions separately (n = 2 missing data) according to Verburgh L, Scherder EJ a, van Lange P a M, Oosterlaan J. Executive functioning in highly talented soccer players. PLoS One. 2014;9: e91254. (DOCX) [file pone.0144395.s003.docx]

**Table S3. Working memory in the TBI and TC groups.**

|  | Groups | |  | Contrasts | |
| --- | --- | --- | --- | --- | --- |
|  | TBI | TC |  | P | Cohen’s *d* |
| *n* | 101 | 44 |  |  |  |
| *Working memory* |  |  |  |  |  |
| Digit Span Forward-Backward difference^a^, M (SD) | 21.0 (13.6) | 22.5 (15.0) |  | .56 | -0.10 |

*Note.* TBI = traumatic brain injury; TC = trauma control; M = mean; SD = standard deviation.

^a^Digit Span score calculated as the number of correct responses multiplied by the maximum correct span, in the forward and backward conditions separately (n=2 missing data) according to Verburgh L, Scherder EJ a, van Lange P a M, Oosterlaan J. Executive functioning in highly talented soccer players. PLoS One. 2014;9: e91254.
