# Supplementary material for: Impaired Visual Integration in Children with Traumatic Brain Injury: An Observational Study
Source: PLoS One. 2015 Dec 4;10(12):e0144395. doi: 10.1371/journal.pone.0144395 (PMC4670090; doi:10.1371/journal.pone.0144395)
Supplement: S4 Table — Note. M = mean; SD = standard deviation. (DOCX) [file pone.0144395.s004.docx]

**Table S4. Non-decision time during the Visual Integration Test in the whole sample.**

|  | Whole Sample |
| --- | --- |
| *n* | 147 |
| *Diffusion Model* |  |
| *Non-decision time* |  |
| Identification, M(SD) | 0.39 (0.12) |
| Localization, M(SD) | 0.38 (0.12) |
| Integration, M(SD) | 0.51 (0.15) |

*Note.* M = mean; SD = standard deviation.
